# Immunometabolic alteration of CD4^+^ T cells in the pathogenesis of primary Sjögren’s syndrome

**DOI:** 10.1007/s10238-024-01429-6

**Published:** 2024-07-22

**Authors:** Yingying Chen, Xuan Luo, Chuiwen Deng, Lidan Zhao, Hui Gao, Jiaxin Zhou, Linyi Peng, Huaxia Yang, Mengtao Li, Wen Zhang, Yan Zhao, Yunyun Fei

**Affiliations:** 1 Department of Rheumatology and Clinical Immunology, Peking Union Medical College Hospital, Chinese Academy of Medical Sciences and Peking Union Medical College, Beijing, 100730 China; 2 National Clinical Research Center for Dermatologic and Immunologic Diseases (NCRC-DID), Ministry of Science and Technology, Beijing, 100730 China; 3 State Key Laboratory of Complex Severe and Rare Diseases, Peking Union Medical College Hospital, Chinese Academy of Medical Science and Peking Union Medical College, Beijing, 100730 China; 4 Key Laboratory of Rheumatology and Clinical Immunology, Ministry of Education, Beijing, 100730 China; 5 Department of Rheumatology and Clinical Immunology, Beijing Chaoyang Hospital, Capital Medical University, Beijing, China; 6 Department of Rheumatology, Beijing Children’s Hospital, Capital Medical University, National Center for Children’s Health, Beijing, 100045 China; 7 Department of Rheumatology and Immunology, Peking University International Hospital, Beijing, China; 8 Department of Health Medicine, Peking Union Medical College Hospital (Dongdan Campus), Chinese Academy of Medical Science, No. 1 Shuaifuyuan Wangfujing, Dongcheng District, Beijing, 100730 China

**Keywords:** Primary Sjögren’s syndrome, Aerobic glycolysis, CD4^+^ T cells, Lactate dehydrogenase A, Reactive oxygen species

## Abstract

Primary Sjögren’s syndrome (pSS) is a prevalent autoimmune disorder wherein CD4^+^ T cells play a pivotal role in its pathogenesis. However, the underlying mechanisms driving the hyperactivity of CD4^+^ T cells in pSS remain poorly understood. This study aimed to investigate the potential role of immunometabolic alterations in driving the hyperactivity of CD4^+^ T cells in pSS. We employed Seahorse XF assay to evaluate the metabolic phenotype of CD4^+^ T cells, conducted flow cytometry to assess the effector function and differentiation of CD4^+^ T cells and measured the level of intracellular reactive oxygen species (ROS). Additionally, transcriptome sequencing, PCR, and Western blotting were utilized to examine the expression of glycolytic genes. Our investigation revealed that activated CD4^+^ T cells from pSS patients exhibited elevated aerobic glycolysis, rather than oxidative phosphorylation, resulting in excessive production of IFN-γ and IL-17A. Inhibition of glycolysis by 2-Deoxy-D-glucose reduced the expression of IFN-γ and IL-17A in activated CD4^+^ T cells and mitigated the differentiation of Th1 and Th17 cells. Furthermore, the expression of glycolytic genes, including *CD3E, CD28, PIK3CA, AKT1, mTOR, MYC, LDHA, PFKL, PFKFB3, and PFKFB4*, was upregulated in activated CD4^+^ T cells from pSS patients. Specifically, the expression and activity of LDHA were enhanced, contributing to an increased level of intracellular ROS. Targeting LDHA with FX-11 or inhibiting ROS with N-acetyl-cysteine had a similar effect on reversing the dysfunction of activated CD4^+^ T cells from pSS patients. Our study unveils heightened aerobic glycolysis in activated CD4^+^ T cells from pSS patients, and inhibition of glycolysis or its metabolite normalizes the dysfunction of activated CD4^+^ T cells. These findings suggest that aerobic glycolysis may be a promising therapeutic target for the treatment of pSS.

**Supplementary Information:**

The online version contains supplementary material available at 10.1007/s10238-024-01429-6.

## Introduction

Primary Sjögren’s syndrome (pSS) is a systemic autoimmune disease characterized by lymphocytic infiltration of the exocrine glands, leading to glandular dysfunction and resulting in symptoms such as xerostomia and keratoconjunctivitis sicca [1–3]. Additionally, extraglandular organs and systems, including the joints, liver, kidneys, lungs, thyroid, central/peripheral nervous system and hematological system, may also be affected. This can contribute to organ dysfunction and significantly impact the quality of life of patients with pSS [4–7].

The hyperactivity of CD4^+^ T cells, particularly Th1 and Th17 cells, has been reported to play essential roles in the development of pSS. Histopathological examination reveals massive infiltration of activated T cells in the salivary and lacrimal glands of pSS patients [8, 9]. Specifically, Th1 cells are observed to produce elevated levels of IFN-γ and TNF-α, contributing not only to the damage of epithelial cells and glandular dysfunction but also to the activation of other immune cells, especially B cells [10–12]. Additionally, an elevated presence of Th17 cells has been identified in both the peripheral blood and exocrine glands of pSS patients, inducing tissue damage through the secretion of IL-17 and fostering autoreactive B cell responses [13–15].

However, the underlying mechanisms driving the hyperactivity of CD4^+^ T cells in pSS remain partially understood. Immunometabolism plays a critical role in regulating the effector functions of immune cells. Upon activation, naive T cells undergo metabolic reprogramming, favoring aerobic glycolysis (also known as the Warburg effect) over oxidative phosphorylation (OxPhos) to acquire energy and biosynthetic intermediates necessary to support their differentiation and effector function [16, 17]. Effector CD4^+^ T cells, including Th1, Th2, and Th17 cells, exhibit heightened glycolytic activity, while Foxp3^+^ regulatory T cells (Tregs) and long-lived memory T cells display elevated rates of lipid oxidation [18, 19].

Metabolic abnormalities of CD4^+^ T cells have been implicated in the pathogenesis of autoimmune diseases such as rheumatoid arthritis (RA) and systemic lupus erythematosus (SLE) [20–22]. Our previous research demonstrated that both OxPhos and glycolysis were enhanced in activated B cells from pSS patients, and inhibition of OxPhos and glycolysis reduced the proliferation, differentiation and effector function of B cells [23]. Besides, Qi et al. revealed that myeloid-derived suppressor cells exhibited high levels of glycolysis and exerted significant pro-inflammatory effects by modulating the differentiation of CD4^+^ T cells in pSS [24]. However, few studies have investigated the potential role of immunometabolic alterations in the hyperactivity of CD4^+^ T cells from pSS patients. Therefore, in this study, we sought to investigate whether CD4^+^ T cells from pSS patients exhibit metabolic abnormalities and explore their potential contribution to the hyperactivity of these cells.

## Material and methods

### Patients

Seventy-four patients were recruited from the Department of Rheumatology at Peking Union Medical College Hospital between August 2020 and May 2023, all meeting the 2016 American College of Rheumatology/European League Against Rheumatism Classification Criteria for primary Sjögren’s syndrome [25]. Clinical data, including manifestations, laboratory examinations, imaging findings, and histopathological results, were meticulously documented. The demographic and clinical characteristics of the included patients are summarized in Table 1. Additionally, sixty-eight age- and sex-matched individuals were recruited as healthy controls (HCs). Written informed consent was obtained from all participants included in this study.

**Table 1:** Clinical characteristics of patients with pSS included in this study

Patients with pSS (n = 74)	
Age (years old)	45 ± 12
Female (n, %)	72 (97%)
C-reactive protein (mg/L)	0.52 (0.30–0.95)
Erythrocyte sedimentation rate (mm/h)	15.50 (8.75–28.50)
Immunoglobulin G (g/L)	17.50 (14.17–22.01)
Rheumatoid factor (IU/mL)	60 (11–162)
Anti-SSA antibody positivity (n, %)	63 (85%)
Anti-SSB antibody positivity (n, %)	24 (32%)
Treatment naïve (n, %)	46 (62%)

### Cell isolation and in-vitro stimulation

Peripheral blood mononuclear cells were isolated from participants’ whole blood using Ficoll-Paque density gradient centrifugation. Total or naïve CD4^+^ T cells were subsequently purified using CD4 MicroBeads or the Naïve CD4^+^ T Cell Isolation Kit II (Miltenyi Biotec), following the manufacturer’s protocols. The cells were cultured in RPMI-1640 medium supplemented with 100units/mL penicillin/streptomycin and 10% fetal bovine serum. For activation of total CD4^+^ T cells, cells were stimulated with pre-coated anti-CD3 antibody (2 μg/ml), anti-CD28 antibody (4 μg/ml), and interleukin-2 (IL-2) (10 ng/ml) for 72 h. For T helper 1 cell (Th1) polarization, cells were stimulated with pre-coated anti-CD3 antibody (2 μg/ml), anti-CD28 antibody (4 μg/ml), IL-2 (10 ng/ml), IL-12 (50 ng/ml), and anti-human IL-4 antibody (10 μg/ml) for 5 days. For Th17 polarization, cells were stimulated with pre-coated anti-CD3 antibody (2 μg/ml), anti-CD28 antibody (4 μg/ml), IL-2 (10 ng/ml), IL-6 (50 ng/ml), IL-1β (10 ng/ml), IL-23 (10 ng/ml), TGF-β (10 ng/ml), anti-human interferon-γ (IFN-γ) antibody (10 μg/ml), and anti-human IL-4 antibody (10 μg/ml) for 5 days. In the inhibition assay, cells were treated with 2-Deoxy-D-glucose (2-DG, 2 mM), metformin (10 mM), rapamycin (10 nM), FX-11 (20 μM) and N-Acetyl-L-Cysteine (NAC, 10 mM), respectively, with the same volume of vehicle added in the control group.

### Metabolic measurement

Activated CD4^+^ T cells (5 × 10^5 cells per well) were seeded onto Seahorse Extracellular Flux (XF) 24 plates (Seahorse biosciences) precoated with Cell-Tak (Corning) and maintained in an unbuffered assay medium (Seahorse biosciences) in a non-CO_2_ incubator at 37 °C for 1 h. The metabolic phenotype of CD4^+^ T cells was assessed using Seahorse XFe24 Extracellular Flux Analyzers (Seahorse biosciences). For the glycolysis stress test, the extracellular acidification rate (ECAR) was measured to indicate the level of glycolysis. Glucose (10 mM), oligomycin (1 μM), and 2-DG (50 mM) were sequentially added during the measurement. For the mitochondrial stress test, the oxygen consumption rate (OCR) was measured to reflect the level of OxPhos. Oligomycin (1.5 μM), Carbonyl cyanide-p-trifluoromethoxyphenylhydrazone (FCCP, 1.5 μM), and Rotenone/Antimycin A (Rot/AA) (0.5 μM) were supplemented in sequence. The supernatant of the cell culture was collected, and extracellular lactate acid was quantified using the L-Lactate Assay kit (Abcam), following the manufacturer’s instructions.

### Flow cytometry

For cell surface staining, cells were incubated in the dark with APC-Cy7-conjugated anti-CD3, for 30 min at 4 °C. For intracellular staining, the cells were fixed and permeabilized using the Foxp3/Transcription Factor Staining Buffer Set (eBiosciences) and then stained with PE-conjugated anti-IL-17A (BioLegend, USA) and PerCP-Cy5.5-conjugated anti-IFN-γ (BioLegend, USA) monoclonal antibodies for 1 h at 4 °C. Subsequently, flow cytometric analysis was performed using the FACSAria™ II flow cytometer (BD Biosciences, San Jose, CA, USA).

As for the gating strategy, magnetically sorted CD4^+^ T cells were initially isolated based on forward scatter-area (FSC-A) and side scatter-area (SSC-A) to exclude debris and dead cells. Specifically, cells were gated within the FSC-A vs. SSC-A plot to exclude small particles, cellular debris and dead cells located at the lower end of both FSC and SSC axes. Further discrimination against doublets was achieved using SSC-H versus SSC-A gating to ensure single-cell analysis. CD4^+^ T cells were subsequently identified by gating on PE-positive and PERCPCY5.5-positive cells to delineate IL-17A and IFN-γ expression within the CD4^+^ T cell population (shown in Fig.S1A–E).

### RNA sequencing (RNA-seq) and data analysis

Activated CD4^+^ T cells were collected from 3 patients with pSS and 3 HCs. Total RNA was extracted using TRIzol reagent (Invitrogen, USA) for subsequent RNA-seq conducted by Novogene (China). Raw data in fastq format were initially processed through in-house scripts, and clean data were obtained by removing reads containing adapters, poly-N sequences, and low-quality reads. HTseq v.0.6.0 was employed to quantify the reads mapped to each gene. Differential expression analysis was carried out using the DESeq2 R package (version 1.10.1). The resulting *P*-values were adjusted using the Benjamini and Hochberg’s method to control the false discovery rate. Genes with an adjusted *P*-value < 0.05 as determined by DESeq2 were considered differentially expressed (DE).

### Real-time quantitative polymerase chain reaction (RT-qPCR)

Total RNA was extracted using the RNA-Quick Purification Kit (EScience Biotech, China) and subjected to reverse transcription using PrimeScript RT Master Mix (Takara). RT-qPCR was conducted with SYBR Premix Ex Taq II (Tli RNaseH Plus, Takara) using the Roche LightCycler 480II system. The primer sequences are listed in Table 2.

**Table 2:** List of RT-qPCR primer sequences

Gene name	Primer sequence
*β-actin* (human)	Forward 5-TGACGTGGACATCCGCAAAG-3 Reverse 5-CTGGAAGGTGGACAGCGAGG-3
*mTOR* (human)	Forward 5’-ACTGGAACCTACCTTTGGCTT-3’ Reverse 5’-ACTGTCTTCATCCGATCCTTCA-3’
*MYC* (human)	Forward 5’-CATCAGCACAACTACGCAGC-3’ Reverse 5’-GCTGGTGCATTTTCGGTTGT-3’
*PFKFB3* (human)	Forward 5′-CAGTTGTGGCCTCCAATATC-3’ Reverse5′-GGCTTCATAGCAACTGATCC-3’
*PFKFB4* (human)	Forward 5′-GCCCAGTTCATCAGTGACCA-3’ Reverse 5′-CGCATCGATCTCGTTGAGGA-3’
*PFKL* (human)	Forward 5′-CATCAGCAACAACGTCCCTG-3’ Reverse 5′-GGCCAGGTAGCCACAGTAAC-3’

### Western blotting analysis

Cells were lysed in 1 × RIPA buffer (Solarbio, China) containing protease/phosphatase inhibitors. The BioRad transfer system was utilized, and scanning was performed using the Tanon 5800 Multi-Image system. The primary antibodies used were as follows: LDHA (3582 T, Cell Signaling Technology, diluted 1:1000), phospho-mTOR (p-mTOR, 5536S, Cell Signaling Technology, diluted 1:1000), mTOR (2983S, Cell Signaling Technology, diluted 1:1000), phospho-PI3K (p-PI3K, 4228S, Cell Signaling Technology, diluted 1:1000), PI3K (4257S, Cell Signaling Technology, diluted 1:1000), and Actin (Cat# A228, Sigma, diluted 1:5000).

### Quantification of reactive oxygen species (ROS)

Cells were rinsed with cold phosphate-buffered saline (PBS) and then incubated with 200μL of PBS supplemented with 1 × ROS Detection Reagent Stock Solution (Sigma) in a 5% CO_2_, 37 °C incubator for 30 min. Data acquisition was performed using the FACSAria™ II flow cytometer (BD Biosciences, San Jose, CA, USA).

### Cell viability

The Cell Counting Kit 8 (CCK8) was employed for drug cytotoxicity analysis. Cells (10^5 cells per well) were seeded onto 96-well cell culture plates and cultured for 3 days. Subsequently, 10 µl of water-soluble tetrazolium salt-8 (WST-8) was added to each well, and the cells were further incubated for 4 h at 37 °C in a 5% CO_2_ atmosphere to allow for the WST-8 reaction. Absorbance was then measured at 450 nm.

### Statistical analysis

The data were analyzed using IBM SPSS Statistics for Windows, Version 24.0 (IBM Corp, Armonk, NY). Categorical variables were presented as number (%) while the normality of distribution for continuous variables was assessed using the Shapiro–Wilk test. Normally distributed data were expressed as mean ± standard deviation, whereas non-normally distributed data were presented as median (interquartile range). Student’s t-test was used for normally distributed continuous variables, while the Mann–Whitney U test was applied for non-normally distributed variables. Paired Sample t-tests were performed for comparisons within the same group with and without inhibition. A *p*-value of < 0.05 (*p < 0.05, **p < 0.01, ***p < 0.001) was considered statistically significant. Graphical presentations were generated using GraphPad Prism software Version 7.0 (GraphPad, San Diego, CA).

## Results

### CD4^+^ T cells from pSS patients exhibited higher aerobic glycolysis upon activation

**Fig. 1:**
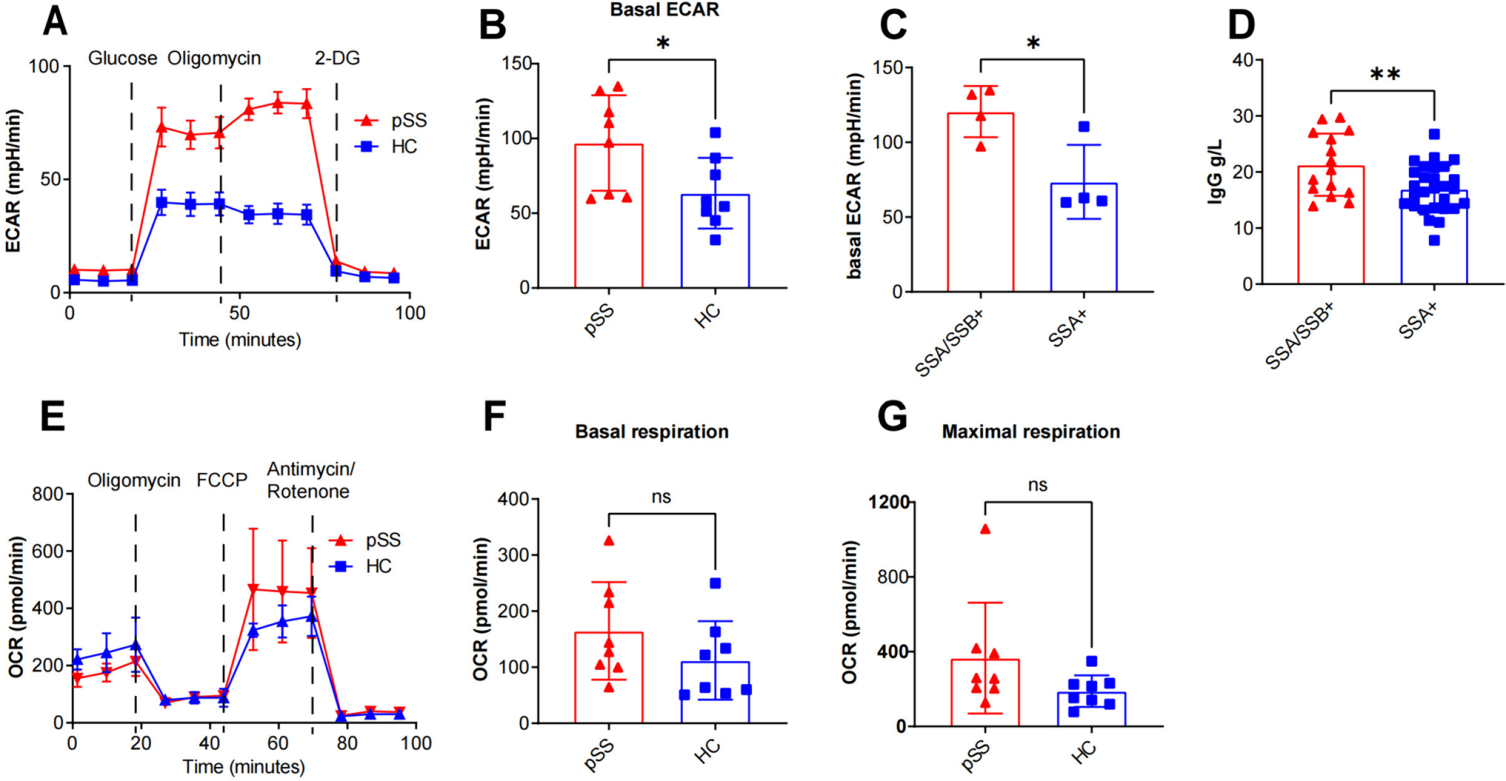
Enhanced glycolytic phenotype in activated CD4^+^ T cells from pSS patients. **A** Representative image depicting the ECAR measured by glycolysis stress test in CD4^+^ T cells. **B** Basal ECAR comparison between CD4^+^ T cells from pSS patients (n = 8) and HCs (n = 8); **C** Basal ECAR comparison between CD4^+^ T cells from patients seropositive for anti-SSA/SSB antibody (SSA/SSB +) (n = 4) and anti-SSA antibody (SSA +) alone (n = 4); **D** Serum IgG comparison between SSA/SSB + patients (n = 15) and SSA + patients (n = 28); **E** Representative image illustrating the OCR measured by MitoStress test in CD4^+^ T cell; **F**, **G** Basal and maximal respiration comparison between CD4^+^ T cells from pSS patients (n = 4) and HCs (n = 4). (Statistical analysis was performed using Student’s t test, *p < 0.05, **p < 0.01, ***p < 0.001)

To delineate the pattern of glucose metabolism in CD4^+^ T cells from pSS patients, we initially compared the ECAR and OCR of activated CD4^+^ T cells between pSS patients and HCs. Upon activation, the basal glycolysis level was notably higher in activated CD4^+^ T cells from pSS patients compared to those from HCs (Fig. 1A, B). This elevation was particularly pronounced in patients seropositive for both anti-SSA and anti-SSB antibodies (SSA/SSB +) compared to those seropositive for anti-SSA antibodies alone (SSA +) (Fig. 1C). Further analysis revealed that SSA/SSB + patients exhibited higher levels of serum IgG than SSA + patients (Fig. 1D). However, no differences were observed between the two groups in terms of OxPhos levels, including basal respiration and maximal respiration (Fig. 1E–G).

### Inhibiting glycolysis decreased the effector function and differentiation of CD4^+^ T cells from pSS patients

**Fig. 2:**
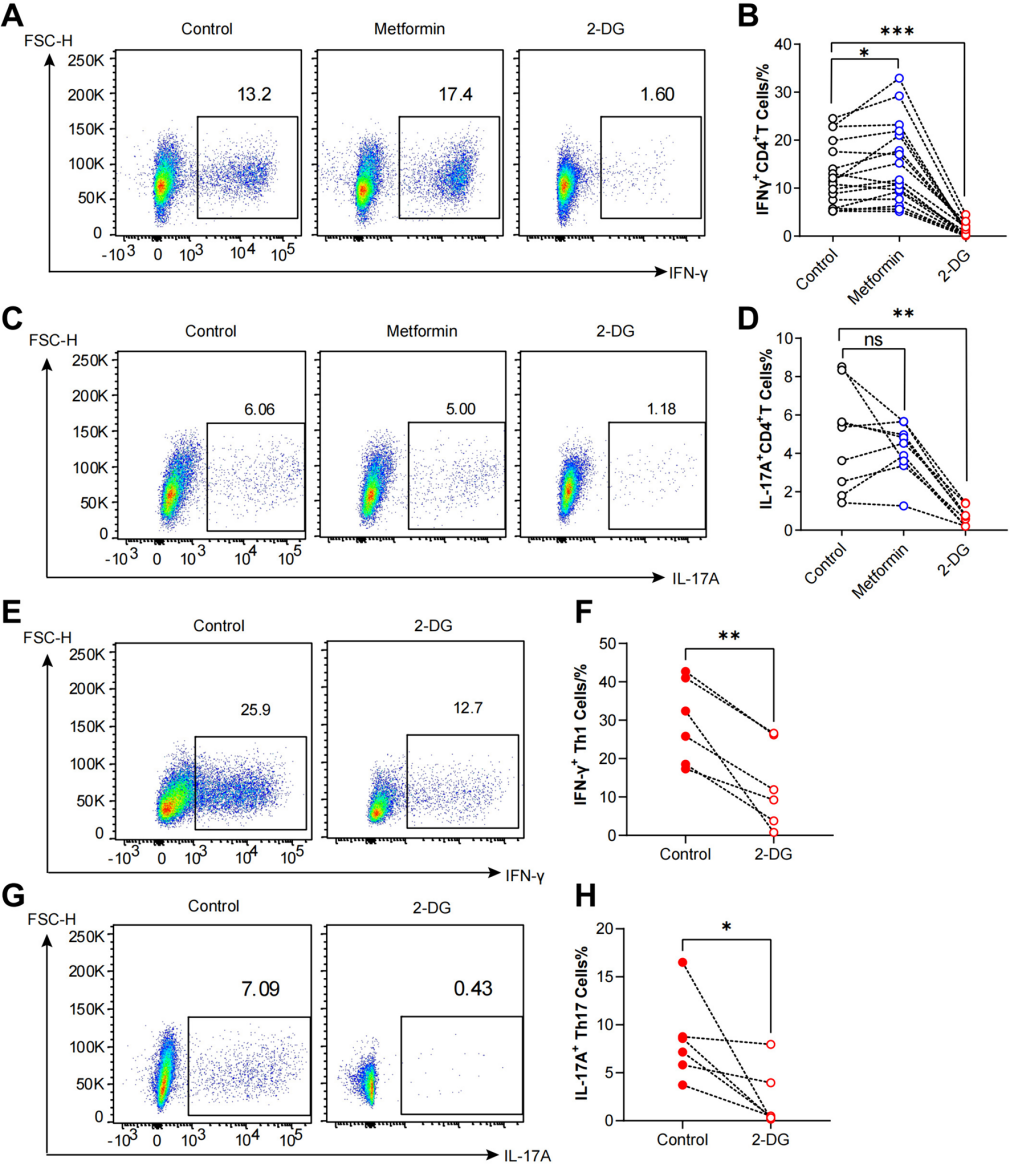
Inhibition of glycolysis reduced the effector function and differentiation of CD4^+^ T cells from pSS patients. **A** Representative images of IFN-γ^+^CD4^+^ T cells in control, metformin and 2-DG groups. **B** Comparison of the percentage of IFN-γ^+^CD4^+^ T cells in different groups (n = 17). **C** Representative images of IL-17A^+^CD4^+^ T cells in control, metformin and 2-DG groups. **D** Comparison of the percentage of IL-17A^+^CD4^+^ T cells in in different groups (n = 9). **E** Representative images of IFN-γ^+^ Th1 cells in control and 2-DG groups. **F** Comparison of the percentage of IFN-γ^+^ Th1 cells in control and 2-DG groups (n = 6). **G** Representative images of IL-17A^+^ Th17 cells in control and 2-DG groups. **H** Comparison of the percentage of IL-17A^+^ Th17 cells in control and 2-DG groups (n = 6). (Statistical analysis between the control and treated group was performed using Pared Sample t test, *p < 0.05, **p < 0.01, ***p < 0.001)

It is widely recognized that effector CD4^+^ T cells primarily rely on aerobic glycolysis for their function. To investigate whether the enhanced glycolysis of CD4^+^ T cells from pSS patients contributed to their hyperactivity, we detected and compared the effector function of activated CD4^+^ T cells from pSS patients and HCs. Results showed that activated CD4^+^ T cells from pSS patients exhibited heightened production of IFN-γ (Fig. S2A, B) and IL-17A (Fig. S2D, E) in contrast with HCs. Interestingly, SSA/SSB + patients displayed a higher percentage of IFN-γ^+^CD4^+^ T cells than SSA + patients (Fig. S2C), consistent with the heightened glycolytic activity observed in SSA/SSB + patients. To delve deeper into the role of elevated aerobic glycolysis in driving hyperactivity of pathogenic CD4^+^ T cells in pSS patients, we utilized 2-DG to target glycolysis, and metformin to inhibit mitochondrial metabolism. Activated CD4^+^ T cells were treated with either 2-DG or metformin, followed by flow cytometry analysis to assess IFN-γ and IL-17A expression. As anticipated, 2-DG significantly inhibited the production of IFN-γ and IL-17A, whereas metformin did not yield the same effect (Fig. 2A-D). Subsequently, naïve CD4^+^ T cells were isolated and induced to differentiate into Th1 and Th17 cells in vitro, with or without 2-DG treatment. Remarkably, inhibition of glycolysis by 2-DG markedly impeded the differentiation of naïve CD4^+^ T cells into Th1 and Th17 cells (Fig. 2E-H). These findings collectively illustrated that the hyperactivation of CD4^+^ T cells in pSS patients was primarily sustained by aerobic glycolysis rather than OxPhos.

### The upregulation of glycolysis was mediated by the activation of the CD28/PI3K/Akt/mTOR pathway

**Fig. 3:**
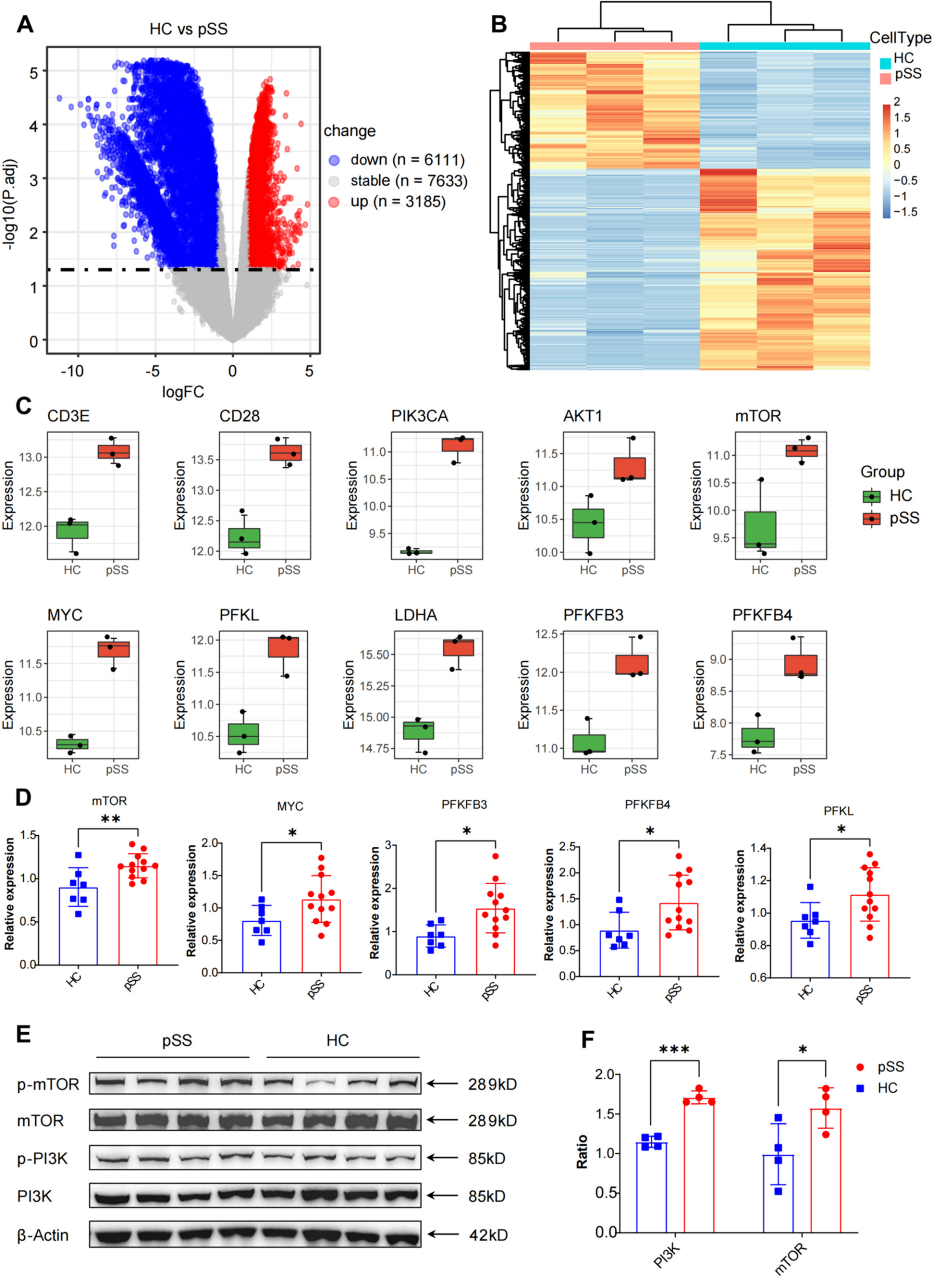
The elevation of glycolysis was mediated by the activation of CD28/PI3K/Akt/mTOR pathway. **A** Volcano plots depicting the differential expression of down-regulated and up-regulated genes in CD4^+^ T cells from pSS patients (n = 3) and HCs (n = 3). **B** Hierarchical clustering heatmap illustrating the gene expression profiles of CD4^+^ T cells from pSS patients (n = 3) and HCs (n = 3). **C** Box plots demonstrating the up-regulation of glycolytic genes in pSS (n = 3) compared to HCs (n = 3). **D** Comparison of the expression of mTOR, MYC, PFKFB3, PFKFB4 and PFKL in pSS (n = 12) and HCs (n = 7) by RT-qPCR. **E** Western blots displaying the expression levels of phosphorylated and total mTOR and PI3K in pSS patients (n = 4) and HCs(n = 4). **F** Comparison of the ratio of phosphorylated to total mTOR and PI3K expression in pSS patients (n = 4) and HCs (n = 4) (Statistical analysis was performed using Student’s t test, NS: not significant, *p < 0.05, **p < 0.01, ***p < 0.001)

We conducted RNA-seq analysis on activated CD4^+^ T cells derived from 3 pSS patients and 3 HCs to delve into the molecular mechanisms underlying the elevated aerobic glycolysis in CD4^+^ T cells from pSS patients. Our analysis identified a total of 3185 upregulated and 6111 downregulated genes in activated CD4^+^ T cells from pSS patients compared to HCs (Fig. 3A). Notably, the gene expression profile of activated CD4^+^ T cells from pSS patients exhibited distinct clustering patterns from that of HCs, as depicted in the hierarchical clustering heatmap (Fig. 3B). Further examination of glycolytic genes unveiled significant upregulation of *CD3E, CD28, PIK3CA, AKT1, mTOR, MYC, PFKL, PFKFB3, PFKFB4, and LDHA* in activated CD4^+^ T cells from pSS patients compared to HCs (Fig. 3C). Subsequently, validation through RT-qPCR confirmed higher expression levels of *mTOR, MYC, PFKFB3, PFKFB4*, and *PFKL* in activated CD4^+^ T cells isolated from an additional 12 pSS patients compared to 7 HCs (Fig. 3D). Furthermore, Western blotting assays validated the upregulation of phospho-mTOR and phospho-PI3K in activated CD4^+^ T cells from pSS patients (Fig. 3E–F). Notably, inhibition of mTOR by rapamycin effectively impaired the secretion of IFN-γ and IL-17A without affecting cell viability (Fig. S3A-D). These findings collectively underscored that the augmented glycolysis in activated CD4^+^ T cells from pSS patients was mediated through the CD28/PI3K/AKT/mTOR pathway, contributing to the hyperfunction of CD4^+^ T cells.

### LDHA-induced ROS enhanced proinflammatory function in activated CD4^+^ T lymphocytes from pSS patients

**Fig. 4:**
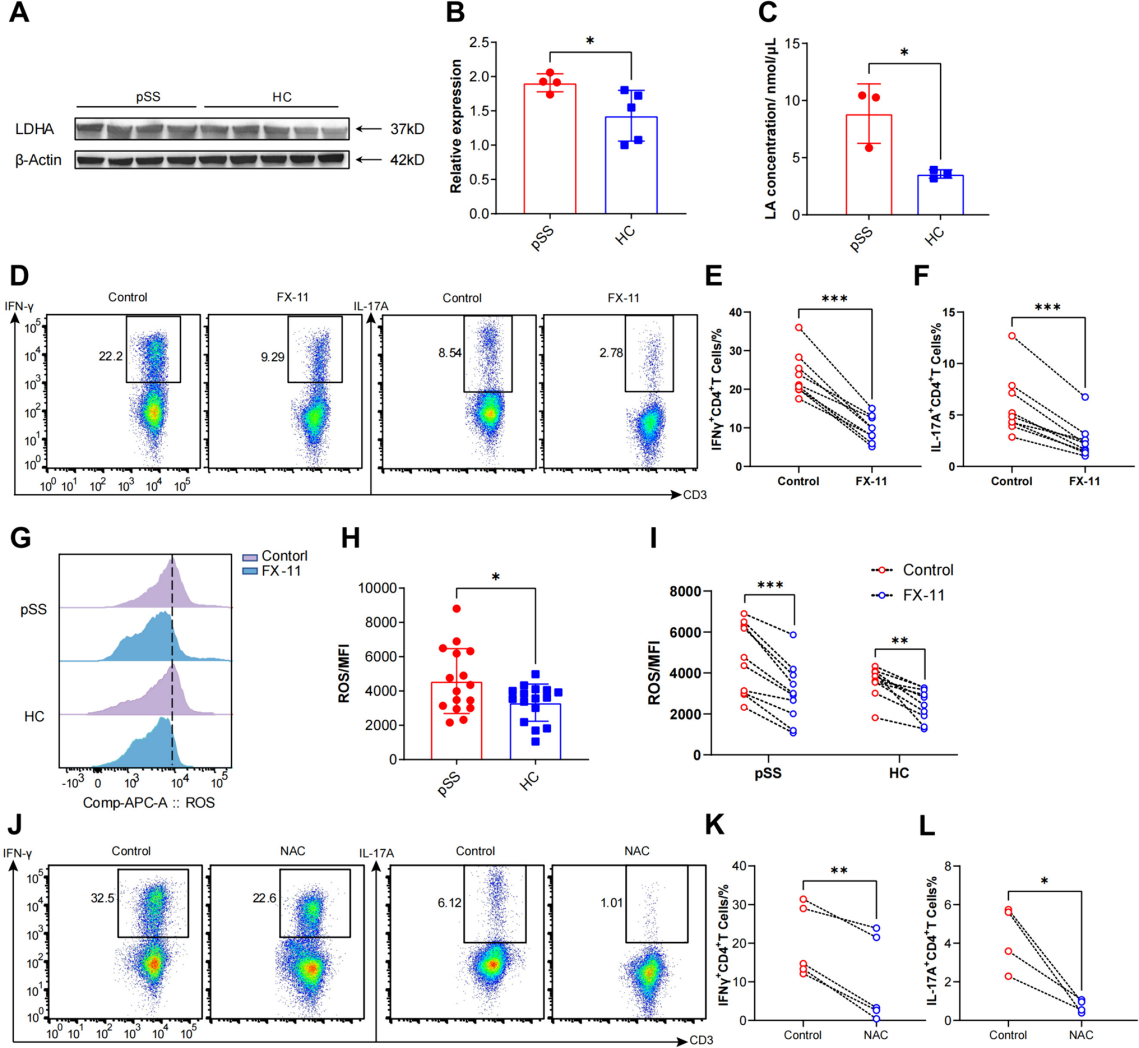
Excessive ROS generated by LDHA promoted the proinflammatory function of activated CD4^+^ T cells in pSS patients. **A** Western blots showing the expression of LDHA in pSS patients (n = 4) and HCs (n = 5). **B** Comparison of the relative expression of LDHA in pSS patients (n = 4) and HCs (n = 5). **C** Comparison of the concentration of lactate acid in culture supernatant from pSS patients (n = 3) and HCs (n = 3). **D** Representative images of IFN-γ^+^CD4^+^ and IL-17A^+^CD4^+^ T cells in pSS with/without FX-11. **E** Comparison of the percentage of IFN-γ^+^CD4^+^ T cells in pSS with/without FX-11 (n = 10). **F** Comparison of the percentage of IL-17A^+^CD4^+^ T cells in pSS with/without FX-11 (n = 10). **G** Representative images of ROS in CD4^+^ T cells from pSS and HCs with/without FX-11. **H** Comparison of ROS levels in CD4^+^T cells from pSS patients (n = 16) and HCs (n = 16). **I** Comparison of ROS levels in CD4^+^ T cells from pSS patients (n = 10) and HCs (n = 10) treated with/without FX-11. **J** Representative images of IFN-γ^+^CD4^+^ and IL-17A^+^CD4^+^ T cells in pSS with/without NAC. **K** Comparison of the percentage of IFN-γ^+^CD4^+^ T cells in pSS with/without NAC (n = 5). **L** Comparison of the percentage of IL-17A^+^CD4^+^ T cells in pSS with/without NAC (n = 5). (Statistical analysis between pSS and HC was performed using Student’s t test, and statistical analysis between control and treated group was performed using Pared Sample t test, NS: not significant, *p < 0.05, **p < 0.01, ***p < 0.001)

According to the results of RNA-seq, LDHA, the key enzyme involved in glycolysis, was found to be overexpressed in activated CD4^+^ T cells from pSS patients, which was further validated by immunoblotting (Fig. 4A, B). Consistently, the culture supernatant of CD4^+^ T cells from pSS patients exhibited a higher level of lactate acid (LA) compared to that from HCs (Fig. 4C). Furthermore, we treated activated CD4^+^ T cells with FX-11, a well-known inhibitor of LDHA, and observed a significant reduction in the production of IFN-γ and IL-17A without affecting cell viability (Fig. 4D–F).

ROS play a pivotal role in the activation and effector function of CD4^+^ T cells, and LDHA can generate ROS by binding to NADH. We detected a significantly higher level of ROS in activated CD4^+^ T cells from pSS patients compared to those from HCs, which could be downregulated by FX-11 (Fig. 4G-I). To explore whether inhibiting LDHA could diminish the production of IFN-γ and IL-17A by reducing the level of ROS, we treated activated CD4^+^ T cells with the ROS inhibitor NAC and assessed the proinflammatory function of activated CD4^+^ T cells. In line with the results of LDHA inhibition, NAC also reduced the production of IFN-γ and IL-17A without affecting cell viability (Fig. 4J-L), suggesting that LDHA might promote the proinflammatory function of activated CD4^+^ T cells through upregulation of ROS.

## Discussion

Our study revealed that activated CD4^+^ T cells from pSS patients exhibited elevated aerobic glycolysis rather than OxPhos, resulting in excessive production of IFN-γ and IL-17A. Further analysis unveiled that enhanced glycolysis was mediated by the CD28/PI3K/AKT/mTOR pathway. Additionally, the expression and activity of LDHA, a key enzyme in glycolysis, were upregulated, leading to the enhanced generation of ROS which might function as signaling molecules regulating the proinflammatory function of activated CD4^+^ T cells from pSS patients.

Metabolic abnormalities of T lymphocytes have been implicated in the pathogenesis of autoimmune diseases. Yin et al. demonstrated that CD4^+^ T cells from lupus-prone mice displayed enhanced glycolysis and OxPhos, and treatment with metformin and 2-DG reduced the production of IFN-γ [22]. Meanwhile, CD4^+^ T cells from SLE patients exhibited metabolic abnormalities similar to that from lupus-prone mice, which indicated that both aerobic glycolysis and OxPhos contributed to the hyperactivity of CD4^+^ T cells in SLE. The role of glycolysis in the CD4^+^ T cells from RA is controversial. Tfh cells from K/BxN mice, an autoimmune model of RA, were highly glycolytic and inhibiting glycolysis in vivo could reduce disease severity [20]. Nonetheless, naive CD4^+^ T cells from RA patients were hypoglycolytic and prone to apoptosis due to deficiency of 6-phosphofructo-2-kinase/fructose-2,6-bisphosphatase 3 (PFKFB3), a key glycolytic enzyme [21]. To date, few studies have identified the pathogenic role of metabolic abnormalities in CD4^+^ T cells from pSS patients. Fu et al. reported that CD4^+^ T cells from SS-like NOD/Ltj mice exhibited enhanced glycolysis and inhibition of glycolysis by 2-DG notably reduced the inflammation of salivary gland and improved the secretary function of submandibular glands of NOD/Ltj mice [26]. Our present study is the first to demonstrate elevated glycolysis in CD4^+^ T cells from pSS patients. Consistent with the hyper-glycolytic phenotype, CD4^+^ T cells from pSS patients exhibited hyperactivity with excessive expression of IFN-γ and IL-17A. Besides, inhibition of glycolysis by 2-DG significantly reduced the production of IFN-γ and IL-17A, demonstrating the significance of glycolysis in driving the hyperactivity of CD4^+^ T cells in pSS.

Upon antigen exposure, TCR/CD28 signaling triggers the activation of downstream PI3K/Akt/mTOR pathway in naïve CD4^+^ T cells, which contributes to the upregulation of glycolytic transcription factors and enzymes and results in the metabolic reprogramming from OxPhos to aerobic glycolysis. The enhanced glycolysis supports the proliferation, effector function, and differentiation of activated CD4^+^ T cells [17, 27–29]. The immune system of pSS is chronically activated by self-antigens, and CD28, a costimulatory molecule providing the second signal for T cell activation, plays a crucial role in the pathogenesis of pSS. Verstappen et al. found that the salivary glands transcriptome of biopsy-positive pSS showed enhanced level of CD3/CD28 T-cell activation signaling in contrast with non-SS sicca patients [30]. Additionally, blocking the interaction between CD86 and CD28 using anti-CD86 antibodies in a murine model for pSS significantly impaired the proliferation of autoantigen-specific T cells [31]. Here, we found notable upregulation of *CD3E*, *CD28*, *PI3K*, *AKT*, and *mTOR* in activated CD4^+^ T cells from pSS patients, indicating that the elevation of glycolysis in CD4^+^ T cells is mediated by the CD28/PI3K/AKT/mTOR pathway. Similar to the effect of 2-DG, inhibiting mTOR with rapamycin decreased the production of IFN-γ and IL-17A in CD4^+^ T cells, further confirming the crucial role of CD28/PI3K/AKT/mTOR-mediated glycolysis in the pathogenesis of pSS.

The upregulation of glycolysis upon activation in CD4^+^ T cells not only provides substrates for biosynthetic pathway, but also plays a more direct role in regulating the effector functions of activated T cells. For instance, GAPDH can serve as RNA binding protein to regulate the translation of IFN-γ and IL-2 mRNA in CD4^+^ T cells [32]. Additionally, at the last step of glycolysis, LDHA can bind to NADH and generates ROS, which serve as intracellular signaling molecules involved in the activation and effector function of CD4^+^ T cells [33–35]. Elevation of ROS in CD4^+^ T cells was previously described in type 1 diabetes, and inhibition of ROS has been shown to attenuate the effector function of CD4^+^ T cells and reduce the development of diabetes [36]. Moreover, oxidative stress, characterized by an imbalance between the production of ROS and physiological antioxidants, has been implicated in the pathogenesis of pSS. Previous studies have demonstrated increased levels of oxidative stress in both plasma and saliva of patients with pSS compared to healthy subjects [37, 38]. In our present study, consistent with elevated glycolysis, we observed higher expression and activity of LDHA in activated CD4^+^ T cells from pSS patients. Furthermore, the level of ROS was also increased, and this elevation was reduced after inhibition of LDHA by FX-11. Additionally, inhibition of ROS also attenuated the expression of IFN-γ and IL-17A, similar to the effect of FX-11 on the effector function of CD4^+^ T cells. These findings provide evidence that ROS generated by the interaction of LDHA and NADH facilitate the proinflammatory function of activated CD4^+^ T cells from pSS patients.

Interestingly, we also observed that the elevation of glycolysis was more pronounced in patients seropositive for both anti-SSA and anti-SSB antibodies compared to those seropositive for anti-SSA antibodies alone. Further analysis revealed that SSA/SSB + patients exhibited a higher percentage of IFN-γ^+^CD4^+^ T cells and serum IgG levels than SSA + patients. Previous study demonstrated that the serum level of β2 microglobulin was significantly elevated in patients seropositive for both anti-SSA and anti-SSB antibodies compared to those seropositive for anti-SSA antibodies alone [39], which suggested heightened immune system activation, particularly in B cell activity, among patients seropositive for both anti-SSA and anti-SSB antibodies. Moreover, excessive expression of IFN-γ in pSS has been shown to promote the activation of B lymphocytes and the production of autoantibodies, ultimately leading to structural destruction and dysfunction of the submandibular gland [40]. Our results provide preliminary evidence suggesting that the upregulation of glycolysis in CD4^+^ T cells might be involved in promoting B cell activity by supporting the effector function of activated CD4^+^ T cells, warranting further investigation.

While our study provides significant insights into the role of glycolysis in the hyperactivity of CD4^+^ T cells from pSS patients, there are several limitations that need to be addressed. Firstly, our study is based on a relatively small sample size, which may limit the generalizability of our findings. Future studies should include larger cohorts to validate our results. Secondly, our study identified that LDHA mediates the hyperactivity of CD4^+^ T cells through the upregulation of ROS. However, we did not thoroughly investigate the precise mechanisms by which ROS contributes to the dysfunction of CD4^+^ T cells. Future research should focus on elucidating these mechanisms to better understand the role of ROS in the pathogenesis of pSS. Additionally, our study relied on in vitro experiments to demonstrate the effects of glycolysis inhibition, and in vivo studies are necessary to confirm the therapeutic potential of targeting glycolysis in pSS.

In conclusion, we have identified elevated aerobic glycolysis in activated CD4^+^ T cells from patients with pSS, which is mediated by enhanced activity of the CD28/PI3K/AKT/mTOR pathway. Apart from providing metabolic support for activated CD4^+^ T cells, our observations suggest that the elevation of aerobic glycolysis may promote the proinflammatory function of activated CD4^+^ T cells through excessive production of ROS, a byproduct of aerobic glycolysis generated by LDHA. ROS can serve as intracellular signaling molecules, regulating the effector function of activated CD4^+^ T cells in patients with pSS. And inhibition of glycolysis reduced the excessive production of IFN-γ and IL-17A in CD4^+^T cells from pSS patients. Thus, targeting the key enzymes or metabolites involved in glycolysis might mitigate inflammation and alleviate disease severity in pSS by normalizing the hyperactivity of CD4^+^ T cells. Overall, our findings indicate that aerobic glycolysis may represent a promising therapeutic target for the treatment of pSS in the future.

## Supplementary Information

Below is the link to the electronic supplementary material.

Supplementary file1 (TIF 16781 KB)

Supplementary file2 (TIF 16831 KB)

Supplementary file3 (TIF 29672 KB)

Supplementary file4 (TIF 18834 KB)

Supplementary file5 (DOCX 13 KB)

## Data Availability

The datasets used and/or analysed during the current study are available from the corresponding author on reasonable request.
