# Supplementary figures and images for: Immunometabolic alteration of CD4+ T cells in the pathogenesis of primary Sjögren’s syndrome
Source: Clin Exp Med. 2024 Jul 22;24(1):163. doi: 10.1007/s10238-024-01429-6 (PMC11263433; doi:10.1007/s10238-024-01429-6)

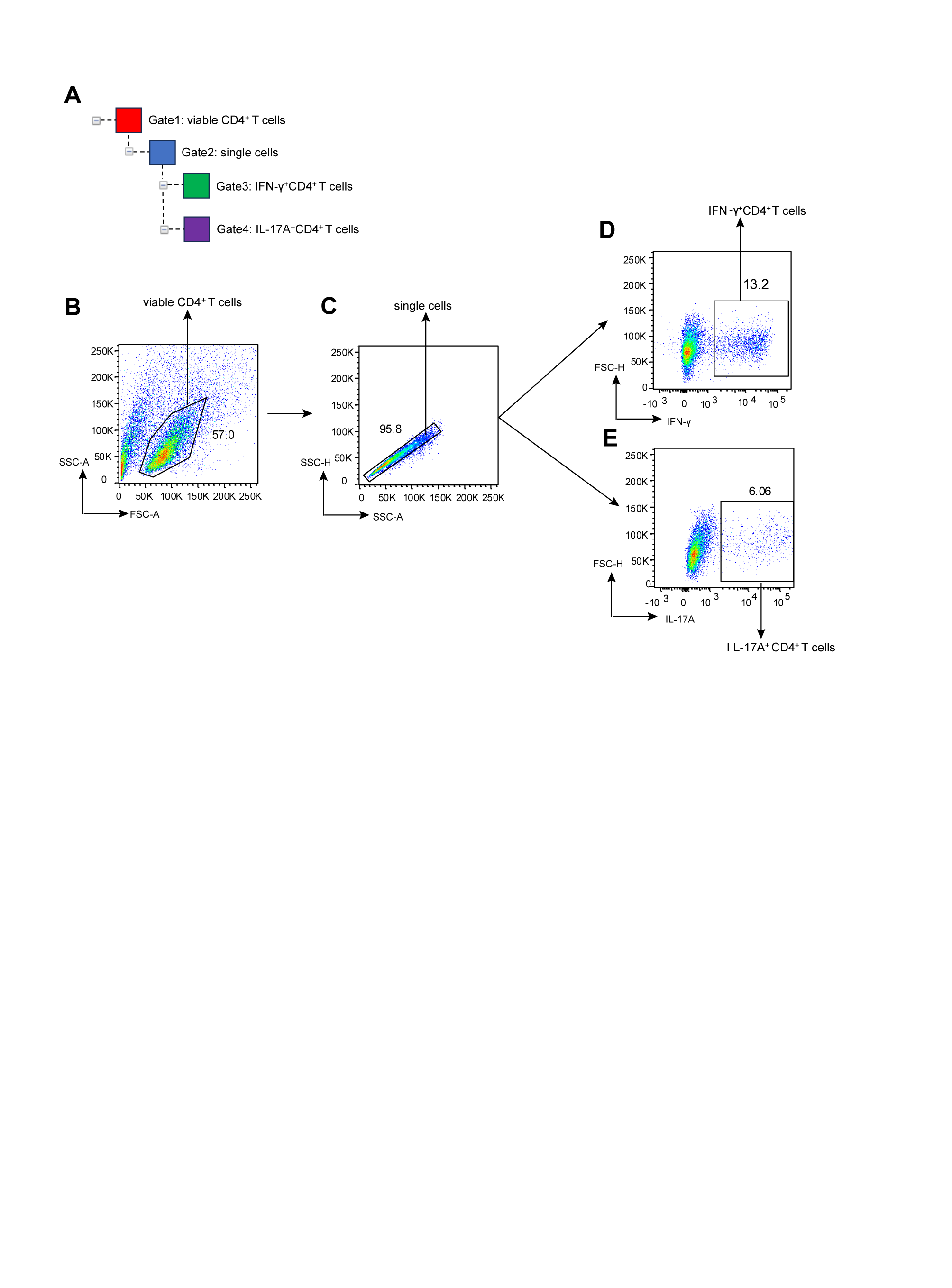

Supplement: Supplementary file 1 — Supplementary file1 (TIF 16781 KB) [file 10238_2024_1429_MOESM1_ESM.tif]

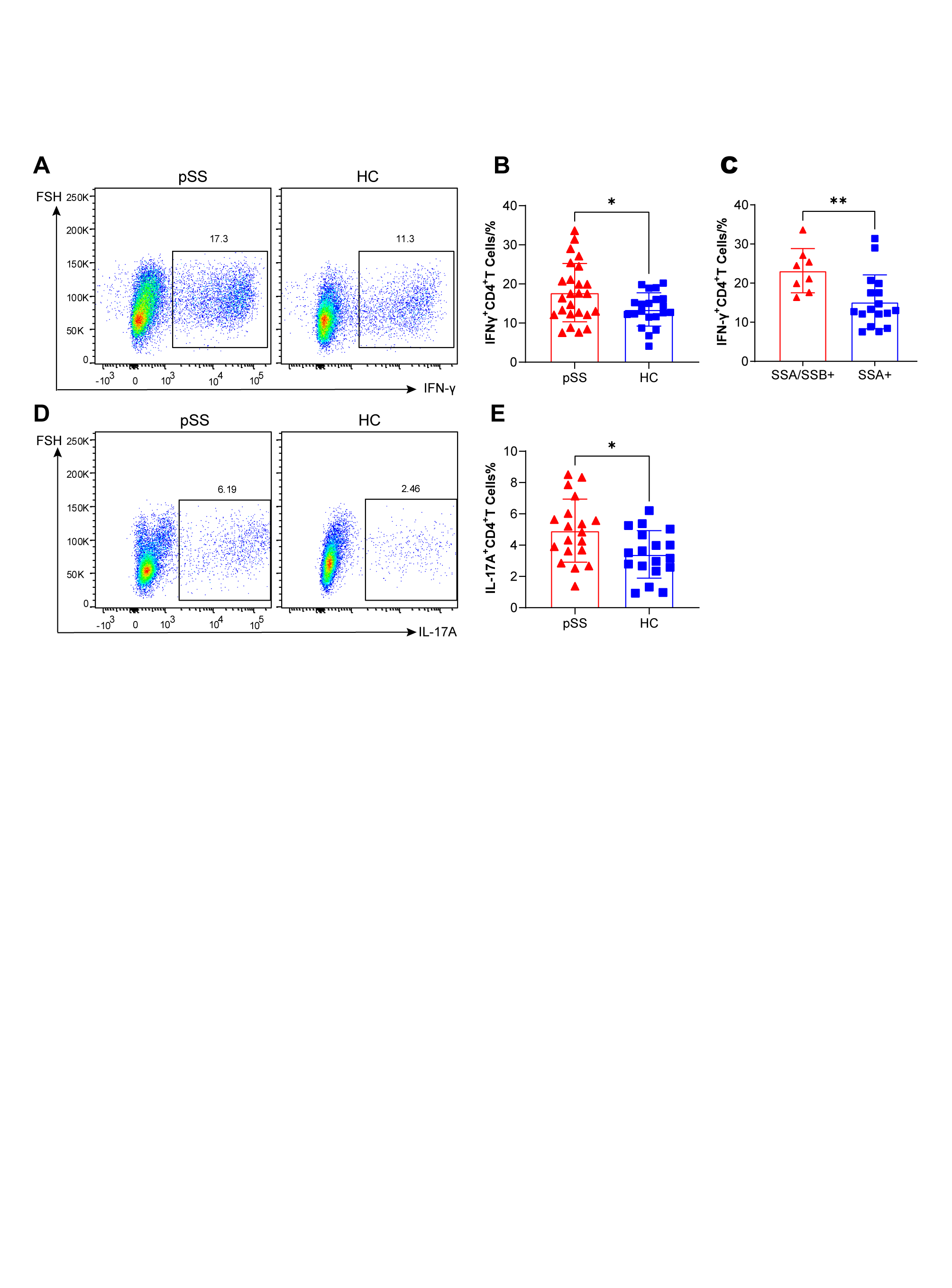

Supplement: Supplementary file 2 — Supplementary file2 (TIF 16831 KB) [file 10238_2024_1429_MOESM2_ESM.tif]

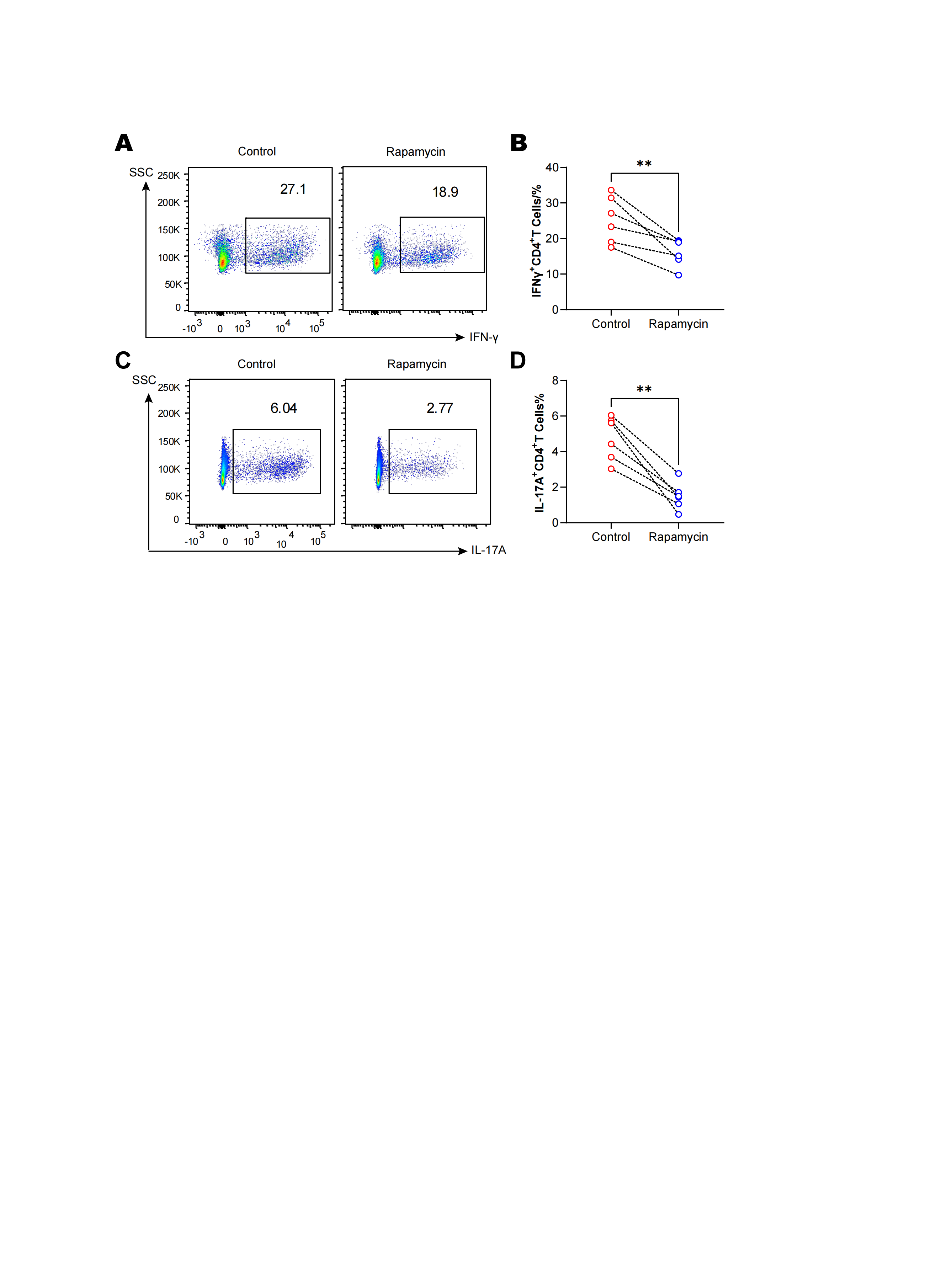

Supplement: Supplementary file 3 — Supplementary file3 (TIF 29672 KB) [file 10238_2024_1429_MOESM3_ESM.tif]

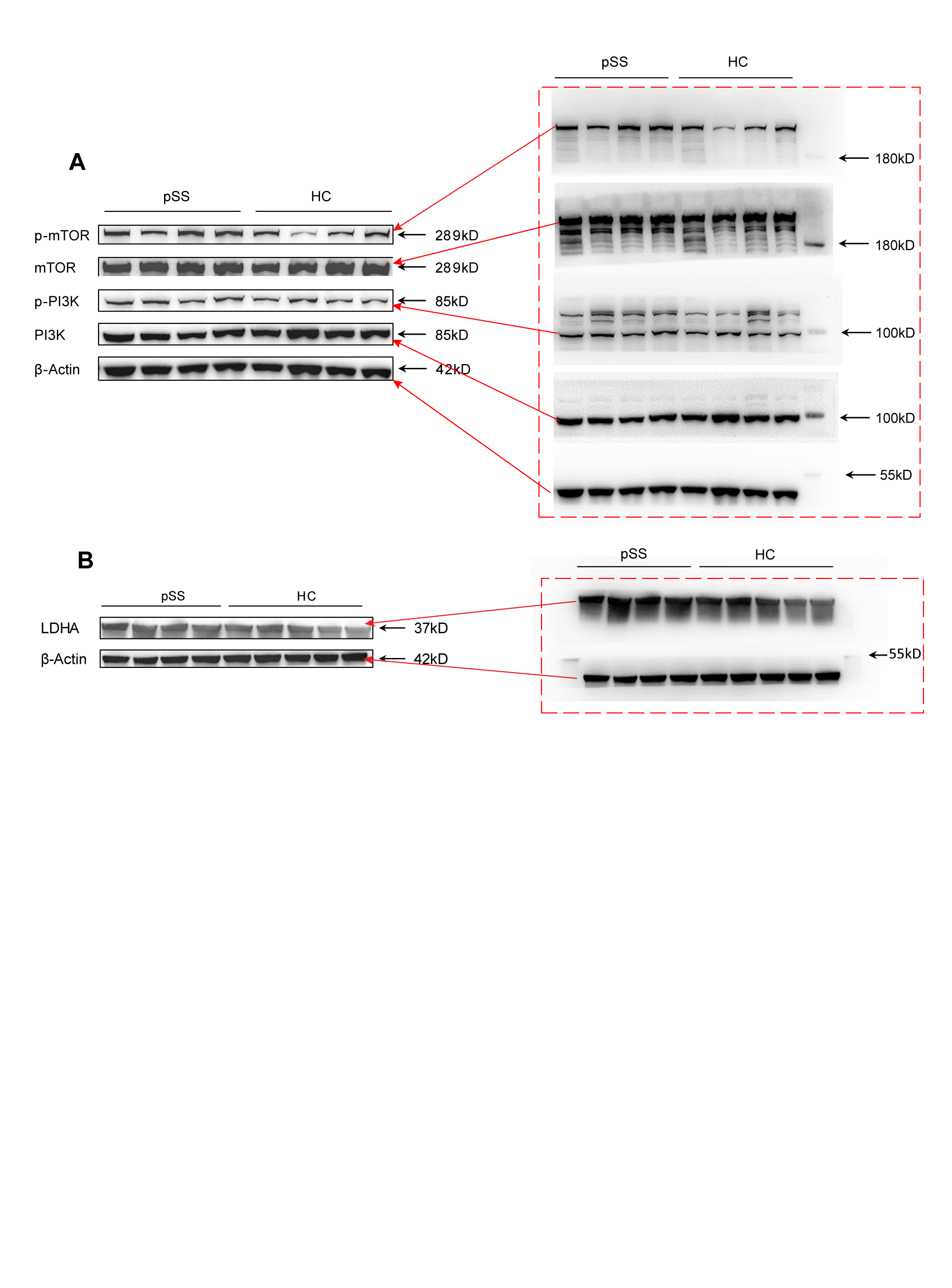

Supplement: Supplementary file 4 — Supplementary file4 (TIF 18834 KB) [file 10238_2024_1429_MOESM4_ESM.tif]
