# Supplementary material for: Immunometabolic alteration of CD4+ T cells in the pathogenesis of primary Sjögren’s syndrome
Source: Clin Exp Med. 2024 Jul 22;24(1):163. doi: 10.1007/s10238-024-01429-6 (PMC11263433; doi:10.1007/s10238-024-01429-6)
Supplement: Supplementary file 5 — Supplementary file5 (DOCX 13 KB) [file 10238_2024_1429_MOESM5_ESM.docx]

Figure S1. Gating hierarchy and representative flow cytometry plots for gating strategy (A) Gating hierarchy of flow cytometry. (B) Initial gating of viable CD4+ T cells based on FSC-A and SSC-A to exclude debris and dead cells. (C) Doublet discrimination using SSC-H vs. SSC-A. (D) IFN-γ expression within the CD4+ T cell population gated on PERCPCY5.5 positivity. (E) IL-17A expression within the CD4+ T cell population gated on PE positivity.

Figure S2. Enhanced glycolysis contributed to hyperactivity of activated CD4+ T cells from pSS patients (A) Representative images of IFN-γ +CD4+ T cells in pSS and HCs. (B) Comparison of frequencies of IFN-γ +CD4+ T cells in pSS (n=25) and HCs (n=21). (C) Comparison of the frequencies of IFN-γ +CD4+ T cells in SSA/SSB+ patients (n=8) and SSA+ patients (n=17). (D) Representative images of IL-17A+CD4+ T cells in pSS and HCs. (E) Comparison of frequencies of IL-17A+CD4+ T cells in pSS (n=19) and HCs (n=18). (Statistical analysis was performed using Student’s t test, NS: not significant, *p < 0.05, **p < 0.01, ***p < 0.001).

Figure S3. Inhibition of rapamycin reduced the effector function of CD4+ T cells from pSS patients (A) Representative images of IFN-γ +CD4+ T cells in rapamycin and control groups. (B) Comparison of the percentage of IFN-γ +CD4+ T cells in rapamycin and control groups (n=6). (C) Representative images of IL-17A+CD4+ T cells in rapamycin and control groups. (D) Comparison of the percentage of IL-17A+CD4+ T cells in rapamycin and control groups (n=6). (Statistical analysis between control and treated group was performed using Pared Sample t test, NS: not significant, *p < 0.05, **p < 0.01, ***p < 0.001)

Figure S4. Full and uncut Western blot images of key proteins analyzed in the study (A) Resentative Western blot images in Figure 3E (left) and the corresponding full and uncut images (right). (B) Representative Western blot images in Figure 4A (left) and the corresponding full and uncut images (right).
